# Uses of Reminiscence in Dementia Care

**DOI:** 10.1093/geroni/igaa057.919

**Published:** 2020-12-16

**Authors:** Abdallah Abu Khait, Juliette Shellman

**Affiliations:** University of Connecticut, Storrs, Connecticut, United States

## Abstract

Improving the quality of life for older adults diagnosed with dementia is a public health research priority. Reminiscence is one non-pharmacological technique used to address the behavioral and psychosocial problems associated with dementia. The uses of reminiscence in dementia care have not yet been integrated, synthesized, critically analyzed, or delineated in the literature. Whittemore and Knafl’s five-step method provided the framework for this integrative review. A comprehensive search of PubMed, CINAHL Plus, SCOPUS, and PsycINFO was undertaken. Articles published in English, focused on participants with a diagnosis of mild to moderate dementia, and with evidence of using reminiscence in dementia care were included. Twenty-six studies published between 2009 and 2019 that met the inclusion criteria were analyzed. Four themes emerged from the integrated findings: (a) recovery from the darkness of depressive symptoms; (b) enhancement of cognitive functions and filling the memory gap; (c) living a fulfilling life in late adulthood, and (d) fulfilling reminiscence functions. The critical appraisal process revealed mixed effectiveness of the use reminiscence on health outcomes in dementia care due to diverse types of reminiscence, different outcomes measures, different data collection toolkits, and a lack of a standardized reminiscence protocol among research studies. Results from this review provide a better understanding of the potential benefits of using reminiscence in dementia care. However, improving the methodological rigor of future studies is necessary to attain conclusive evidence of the effectiveness of using reminiscence in dementia care. Implications of these findings for gerontological education, practice, and research will be presented.

